# Supplementary material for: Design and realization of topological Dirac fermions on a triangular lattice
Source: Nat Commun. 2021 Sep 13;12:5396. doi: 10.1038/s41467-021-25627-y (PMC8438025; doi:10.1038/s41467-021-25627-y)
Supplement: Supplementary file 1 — Supplementary Information [file 41467_2021_25627_MOESM1_ESM.pdf]

Design and realization of topological Dirac fermions on a triangular lattice - Supplementary Information -

Maximilian Bauernfeind,[1,2,*] Jonas Erhardt,[1,2,*] Philipp Eck,[2,3,*] Pardeep K. Thakur,[4] Judith Gabel,[4] Tien-Lin Lee,[4] Jörg Schäfer,[1,2] Simon Moser,[1,2] Domenico Di Sante,[3,5,6] Ralph Claessen,[1,2,†] and Giorgio Sangiovanni[2,3,‡]

[1] *Physikalisches Institut, Universität Würzburg, D-97074 Würzburg, Germany*
[2] *Würzburg-Dresden Cluster of Excellence ct.qmat, Universität Würzburg, D-97074 Würzburg, Germany*
[3] *Institut für Theoretische Physik und Astrophysik, Universität Würzburg, D-97074 Würzburg, Germany*
[4] *Diamond Light Source, Harwell Science and Innovation Campus, Didcot, OX11 0DE, United Kingdom*
[5] *Department of Physics and Astronomy, University of Bologna, 40127 Bologna, Italy*
[6] *Center for Computational Quantum Physics, Flatiron Institute, New York, 10010 NY, USA*

(Dated: September 1, 2021)

TABLE OF CONTENTS

Supplementary Note 1. Overview of triangular and hexagonal adsorbate systems 2

Supplementary Note 2. Low energy tight-binding and Wannier model 3

Supplementary Note 3. Chemical and structural characterization 9

Supplementary Note 4. Band mapping 17

Supplementary Note 5. Local charge distribution and spectroscopy 19

References 25

* These authors have contributed equally
† e-mail: claessen@physik.uni-wuerzburg.de
‡ e-mail: sangiovanni@physik.uni-wuerzburg.de

Supplementary Note 1. OVERVIEW OF TRIANGULAR AND HEXAGONAL ADSORBATE SYSTEMS

In this section, we give an excerpt of surface systems in triangular and hexagonal geometry on various substrates. Among the experimentally realized adsorbate systems only a small fraction of hexagonal systems form on an insulating substrate (Supplementary Fig. 1). However, metallic substrates are detrimental to many applications based on edge state transport in quantum spin Hall insulators (QSHIs). On the contrary, Supplementary Fig. 1 clearly indicates a large abundance of triangular adsorbate systems on insulating substrates. Aiming for an insulating bulk, as necessary for QSHIs, this class of materials constitutes a promising design environment.

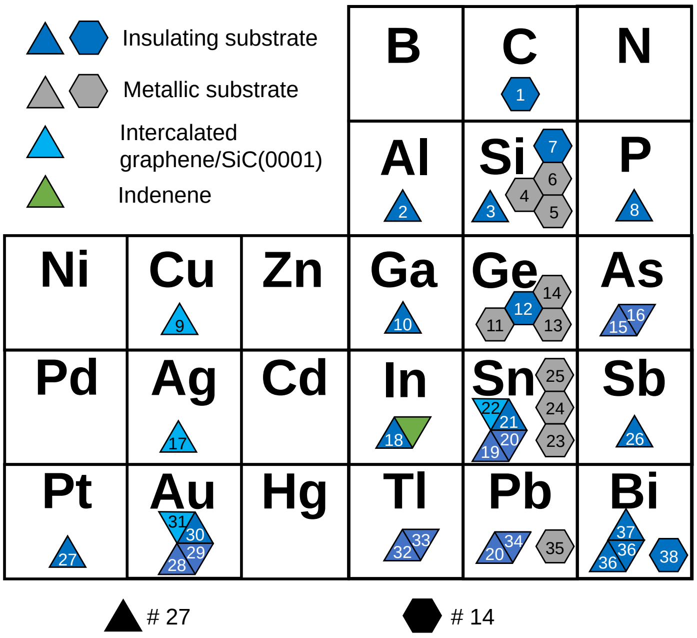

Supplementary Figure 1. Schematic overview of adsorbate systems in the (sub-) monolayer coverage regime, listed according to their adsorption geometry, i.e., triangular vs. hexagonal lattice structure. Adsorbate systems on insulating (metallic) substrates are marked in dark blue (grey). Monolayer systems based on intercalation to the graphene/SiC(0001) interface are considered separately and marked in light blue. We list 27 triangular and 14 honeycomb systems, referenced by the numbers inside the pictograms.

Supplementary Note 2. LOW ENERGY TIGHT-BINDING AND WANNIER MODEL

In this section, we present a step-wise development of the minimal tight-binding model describing the low energy physics of indenene on SiC. The unit cell of indenene is spanned by the lattice vectors $\vec{a}_1 = a(1,0)$ and $\vec{a}_2 = a(-0.5, \sqrt{3}/2)$, with lattice constant a. The Hamiltonian is constructed in terms of a basis of $\{p_x, p_y, p_z\}$ orbitals localized at $(0,0)$ within the unit cell. We consider the local on-site energy H^{LOC}, the nearest neighbor hybridization H^{HYB} (parametrized by Slater-Koster parameters [39]), the atomic spin-orbit coupling (SOC) H^{SOC}, the out-of-plane mirror symmetry breaking via an underlying substrate H^{MIR}, as well as a substrate induced in-plane inversion symmetry breaking (ISB) along nearest neighbors H^{ISB}:

$$H = H^{\mathrm{LOC}} + H^{\mathrm{HYB}} + H^{\mathrm{SOC}} + H^{\mathrm{MIR}} + H^{\mathrm{ISB}}. \tag{1}$$

To model the various scenarios of indenene (freestanding/on substrate), we chose the Slater-Koster parameters V such that the density functional theory (DFT) band structure of the corresponding unit cell is qualitatively reproduced, and summarize the tight-binding parameters in Supplementary Table 1. The electron filling of the p bands is 2.

E_{inpl}	V^{σ}	V^{π}	$V^{\pi}_{p_z}$	λ_{SOC} [1]	λ_{MIR}	Δ_{MIR}
2.10	2.11	0.26	-0.65	0.60	0.7	-2.60

Supplementary Table 1. Tight binding parameters, qualitatively obtained from a Wannier Hamiltonian.

Freestanding triangular lattice

Among the considered systems, the freestanding triangular lattice possesses the highest point group symmetry D_{6h}, with inversion and mirror symmetries preserved. The Hamiltonian reduces to

$$H = H^{\mathrm{LOC}} + H^{\mathrm{HYB}} + H^{\mathrm{SOC}}\ , \tag{2}$$

where the local Hamiltonian is given by

$$H^{\mathrm{LOC}} = \begin{pmatrix} E_{inpl} & 0 & 0 \\ 0 & E_{inpl} & 0 \\ 0 & 0 & E_{p_z} \end{pmatrix}\ , \tag{3}$$

and where E_{inpl} and E_{p_z} are the on-site energies of the p_x, p_y and the p_z orbitals, respectively. In the freestanding case, the on-site energy of the p orbitals is degenerate and we have $E_{inpl} = E_{p_z}$. The nearest neighbor p-p transfer integrals are constructed from the Slater-Koster hopping parameters and depend on the σ/π-hybridization:

$$\langle p_i|H|p_i\rangle = n_i^2 V^{\sigma} + (1-n_i^2)V^{\pi}, \tag{4}$$

$$\langle p_i|H|p_j\rangle = -n_i n_j (V^{\pi} - V^{\sigma}). \tag{5}$$

With $i \in \{x,y,z\}$ and $i \neq j$, the coefficients n_i incorporate the in-plane orientation ($n_x = \cos(\varphi)\sin(\theta)$, $n_y = \sin(\varphi)\sin(\theta)$ and $n_z = \cos(\theta)$) with azimuth angle φ and polar angle θ. The general hopping Hamiltonian thus reads:

$$H^{\mathrm{HYB}} = \begin{pmatrix} V_{p_x p_x}(\mathbf{k}) & V_{p_y p_x}(\mathbf{k}) & V_{p_z p_x}(\mathbf{k}) \\ V_{p_x p_y}(\mathbf{k}) & V_{p_y p_y}(\mathbf{k}) & V_{p_z p_y}(\mathbf{k}) \\ V_{p_x p_z}(\mathbf{k}) & V_{p_y p_z}(\mathbf{k}) & V_{p_z p_z}(\mathbf{k}) \end{pmatrix}. \tag{6}$$

[1] Please note: We have chosen λ_{SOC} twice as large as DFT predicts for a better visualization of the splitting of the Dirac bands.

In the following, we set the lattice constant $a = 1$ and give the crystal momenta in terms of reduced coordinates $\mathbf{k} = \frac{2\pi}{a}(k_1, k_2)$. The matrix elements are given by:

$$V_{p_xp_x}(\mathbf{k}) = 2V^\sigma \cos(k_1) + \frac{V^\sigma + 3V^\pi}{2}(\cos(k_2) + \cos(k_1 + k_2)) \ , \tag{7}$$
$$V_{p_yp_y}(\mathbf{k}) = 2V^\pi \cos(k_1) + \frac{3V^\sigma + V^\pi}{2}(\cos(k_2) + \cos(k_1 + k_2)) \ , \tag{8}$$
$$V_{p_zp_z}(\mathbf{k}) = 2V^\pi_{p_z}(\cos(k_1) + \cos(k_2) + \cos(k_1 + k_2)) \ , \tag{9}$$
$$V_{p_xp_y}(\mathbf{k}) = V_{p_yp_x}(\mathbf{k}) = -\frac{\sqrt{3}}{2}(V^\pi - V^\sigma)(-\cos(k_2) + \cos(k_1 + k_2)) \ , \tag{10}$$
$$V_{p_xp_z}(\mathbf{k}) = V_{p_zp_x}(\mathbf{k}) = V_{p_yp_z}(\mathbf{k}) = V_{p_zp_y}(\mathbf{k}) = 0 \ . \tag{11}$$

Identity 11 reflects the orthogonality of the in-plane and the p_z orbitals, and the block diagonal form of the hybridization matrix (6). The in-plane hybridization matrix elements $V_{p_xp_x}, V_{p_yp_y}$ and $V_{p_xp_y}$ vanish linearly at the K/K′-points $(\pm 1/3, \pm 1/3)$ and give rise to a Dirac-like dispersion.

Local spin-orbit coupling

In contrast to Kane-Mele-type QSHIs, the major contribution of the spin-orbit interaction in indenene on SiC arises from the local (i.e., the **k**-independent) SOC on the indium atoms:

$$H^{\mathrm{SOC}} = \lambda_{\mathrm{SOC}} \vec{L} \cdot \vec{S} \ . \tag{12}$$

Expressing the in-plane orbitals in terms of spherical harmonics

$$|p_x\rangle = \frac{1}{\sqrt{2}}\left(-|1,1\rangle + |1,-1\rangle\right), \qquad |p_y\rangle = \frac{i}{\sqrt{2}}\left(+|1,1\rangle + |1,-1\rangle\right), \tag{13}$$

with angular momentum operators

$$L_x = \frac{1}{2}\left(L_+ + L_-\right), \qquad L_y = -\frac{i}{2}\left(L_+ - L_-\right), \qquad L_z = L_z, \tag{14}$$

the relevant matrix elements gapping the Dirac states at K/K′ are obtained by

$$\langle p_y|\vec{L_z} \cdot \vec{S_z}|p_x\rangle = iS_z, \qquad \langle p_x|\vec{L_z} \cdot \vec{S_z}|p_y\rangle = -iS_z. \tag{15}$$

The low-energy SOC Hamiltonian at K/K′ of the in-plane subspace thus reads

$$H^{\mathrm{SOC}}_{p_xp_y} = \frac{\lambda_{\mathrm{SOC}}}{2}\tau_y \otimes \sigma_z = \frac{\lambda_{\mathrm{SOC}}}{2} L_z \otimes \sigma_z \ , \tag{16}$$

where the Pauli matrix τ_y acts on the orbital degrees of freedom, which is the representation of the out-of-plane angular momentum operator L_z. This SOC Hamiltonian opens a gap of size λ_{SOC}, with valence and conduction states that are characterized by the total angular momentum $j = |j_z| = \frac{1}{2}$ and $\frac{3}{2}$, respectively (see also insets of Fig. 2a-c in the main manuscript).

Out-of plane mirror symmetry breaking

Introducing the substrate breaks the out-of-plane mirror symmetry of indenene (e.g. through an electrical field) and reduces the point group symmetry to C_{6v}. For the sake of simplicity, we assume that H^{HYB} remains unaltered and introduce H^{MIR} to describe the mirror symmetry breaking. The full Hamiltonian can now be written as

$$H = H^{\mathrm{LOC}} + H^{\mathrm{HYB}} + H^{\mathrm{SOC}} + H^{\mathrm{MIR}}. \tag{17}$$

This mirror symmetry breaking activates the hybridization between the in-plane and p_z manifold and lifts the degeneracy of the on-site energies:

$$H^{\mathrm{MIR}} = \begin{pmatrix} 0 & 0 & V_{p_zp_x}(\mathbf{k}) \\ 0 & 0 & V_{p_zp_y}(\mathbf{k}) \\ V_{p_xp_z}(\mathbf{k}) & V_{p_yp_z}(\mathbf{k}) & \Delta_{\mathrm{MIR}} \end{pmatrix} \ . \tag{18}$$

Further, the presence of the mirror symmetry breaking can be interpreted as an out-of-plane displacement of the p_z orbital with respect to the in-plane manifold. Referring to equation 5, the elements of $H^{\rm MIR}$ can be parametrized by the mirror symmetry breaking strength $\lambda_{\rm MIR} = n_{x,y}n_z(V^\pi - V^\sigma)$ and read

$$V_{p_xp_z}(\mathbf{k}) = i\lambda_{\rm MIR}\left[2\sin(k_1) - \sin(k_2) + \sin(k_1+k_2)\right] \ , \tag{19}$$
$$V_{p_yp_z}(\mathbf{k}) = \sqrt{3}i\lambda_{\rm MIR}\left[\sin(k_2) + \sin(k_1+k_2)\right] \ . \tag{20}$$

The mirror symmetry breaking opens hybridization gaps by avoiding band crossings between the in-plane and the out-of-plane p_z orbital manifolds, as shown in Fig. 2b of the main manuscript. At K and K′, the hybridization gap vanishes and the Kramers degeneracy is preserved. Concomitantly taking into account the SOC term additionally opens a gap at K/K′, and a global – topologically non-trivial – gap is obtained.

In-plane inversion symmetry breaking

For indenene on SiC, the top layer carbon substrate atoms break in-plane inversion symmetry and lead to a further reduction of its point group to C_{3v}. We model this effect by a potential peaking at the centers A (1/3, 2/3) and B (2/3, 1/3) of the two in-equivalent triangles spanned by the next-neighbors of the triangular lattice, defined in Fig. 2f of the main manuscript. Restricting the ISB interaction to the in-plane orbitals, we can write the effective Hamiltonian in the (p_x,p_y) subspace:

$$H^{\rm ISB} = \lambda_{\rm ISB}\left(\sin(k_1) + \sin(k_2) - \sin(k_1+k_2)\right)\tau_y \ . \tag{21}$$

The ISB interaction now lifts the degeneracy of the in-plane bands at K and K′, shown in Supplementary Fig. 2. This term has the same representation as the out-of-plane angular momentum operator L_z acting on the (p_x,p_y) subspace and is given by τ_y. Hence, this term promotes an out-of-plane angular momentum polarization with opposite alignment at K and K′ (left panel). If SOC is included, the low-energy Hamiltonian of the (p_x,p_y) bands at K/K′ is given by

$$H({\rm K/K'}) = H^{\rm SOC} + H^{\rm ISB}({\rm K/K'}) \tag{22}$$
$$= \frac{\lambda_{\rm SOC}}{2}\tau_y\otimes\sigma_z \pm \frac{3\sqrt{3}}{2}\lambda_{\rm ISB}\tau_y\otimes\sigma_0 = \lambda_{\rm SOC}L_z\cdot S_z \pm \frac{\sqrt{3}}{2}\lambda_{\rm ISB}L_z = L_z\left(\lambda_{\rm SOC}S_z \pm \frac{3\sqrt{3}}{2}\lambda_{\rm ISB}\right) \ , \tag{23}$$

with the four eigenvalues $E = \pm\frac{1}{2}\lambda_{\rm SOC} \pm \frac{3\sqrt{3}}{2}\lambda_{\rm ISB}$. In the presence of both interactions, the Kramers degeneracy at K/K′ is lifted. If ISB dominates over SOC (Supplementary Fig. 2 middle panel), the $\langle J\rangle$ values of the two valence bands are different, and the system is topologically trivial. If SOC dominates over ISB, i.e., in the limit of large $\lambda_{\rm SOC}$ (rightmost panel of Supplementary Fig. 2), valence and conduction bands share the same $\langle J\rangle$ (1/2 and 3/2, respectively) and the system is topologically non-trivial. The $\langle L_z\rangle$ character then alternates in the valence (conduction) band doublet (main panels of Supplementary Fig. 2).

Charge localization of the Dirac-states

In the presence of ISB and/or SOC, the Dirac-states at K/K′ are described by $|p_\pm\rangle \propto e^{\pm i\phi}$ orbitals. The real space amplitude of a Bloch wave with momentum $\mathbf{k}$ at position r then is given by

$$|\Psi_\pm(\mathbf{k},r)\rangle = \sum_{\mathbf{R}} e^{i\mathbf{k}\cdot\mathbf{R}}|p_\pm(\mathbf{R},r)\rangle \ , \tag{24}$$

where R sums over all lattice sites. By considering the three nearest-neighbor orbitals at (0,0), (0,1) and (1,1) around the center point $A = (1/3, 2/3)$, as well as (0,0), (1,0) and (1,1) for the other center point $B = (2/3, 1/3)$, the squared

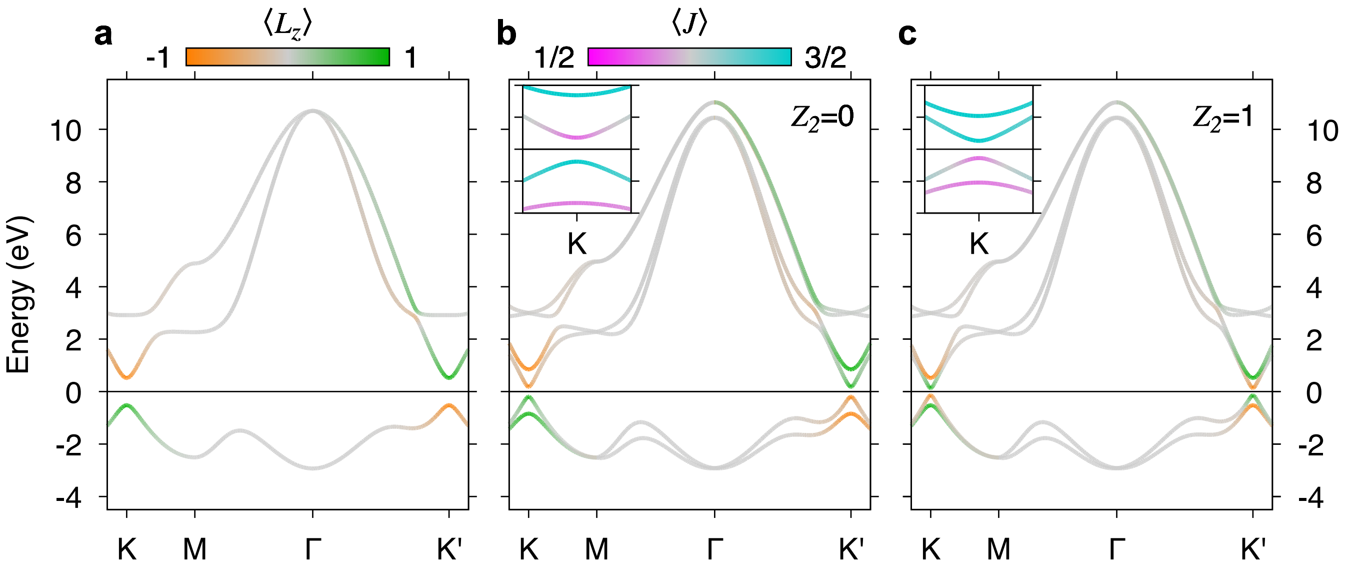

Supplementary Figure 2. **a**, Band structure without SOC and $\lambda_{\text{ISB}} = 0.2$, **b** same Hamiltonian with SOC ($\mathbb{Z}_2 = 0$) and **c** with SOC and $\lambda_{\text{ISB}} = 0.075$ ($\mathbb{Z}_2 = 1$). The ISB opens a hybridization gap and lifts the Kramers degeneracy at K/K$'$. **b**, If $\lambda_{\text{SOC}} < 3\sqrt{3}\lambda_{\text{ISB}}$, ISB dominates over SOC at K/K$'$ and the L_z-quantum number becomes a good quantum number for describing the valence and conduction states, the system is in the topologically trivial phase. **c**, If the system is in the SOC-dominated topologically non-trivial phase, both L_z quantum numbers are present in the valley momenta valence and conduction states.

module of the Bloch wave function at K$= (1/3, 1/3)$ and K$' = -K$ are given by:

$$|\Psi_\pm(\text{K}, A)|^2 \propto \frac{1}{9}|e^{2\pi i\left(0\pm\frac{3}{12}\right)} + e^{2\pi i\left(\frac{1}{3}\pm\frac{11}{12}\right)} + e^{2\pi i\left(\frac{2}{3}\pm\frac{7}{12}\right)}|^2 = \begin{cases} 1, & \text{for } p_+ \\ 0, & \text{for } p_- \end{cases}, \tag{25}$$

$$|\Psi_\pm(\text{K}', A)|^2 \propto \frac{1}{9}|e^{2\pi i\left(0\pm\frac{3}{12}\right)} + e^{2\pi i\left(\frac{1}{3}\pm\frac{11}{12}\right)} + e^{2\pi i\left(\frac{2}{3}\pm\frac{7}{12}\right)}|^2 = \begin{cases} 0, & \text{for } p_+ \\ 1, & \text{for } p_- \end{cases}, \tag{26}$$

$$|\Psi_\pm(\text{K}, B)|^2 \propto \frac{1}{9}|e^{2\pi i\left(0\pm\frac{1}{12}\right)} + e^{2\pi i\left(\frac{1}{3}\pm\frac{5}{12}\right)} + e^{2\pi i\left(\frac{2}{3}\pm\frac{9}{12}\right)}|^2 = \begin{cases} 0, & \text{for } p_+ \\ 1, & \text{for } p_- \end{cases}, \tag{27}$$

$$|\Psi_\pm(\text{K}', B)|^2 \propto \frac{1}{9}|e^{2\pi i\left(0\pm\frac{1}{12}\right)} + e^{2\pi i\left(-\frac{1}{3}\pm\frac{5}{12}\right)} + e^{2\pi i\left(-\frac{2}{3}\pm\frac{9}{12}\right)}|^2 = \begin{cases} 1, & \text{for } p_+ \\ 0, & \text{for } p_- \end{cases}. \tag{28}$$

Further, the orbital angular momentum eigenfunctions p_+ and p_- possess opposite L_z eigenvalues, $+1$ and -1, respectively. As illustrated in Supplementary Fig. 2, both values of L_z characterize the VBs and CBs at K/K$'$ in the non-trivial phase. This is contrary to the trivial phase, where the valence bands at a given valley K/K$'$ share the same L_z. For instance, considering a band with $L_z = 1$ at K and $L_z = -1$ at K$'$, i.e., p_+ and p_-, respectively, the Bloch wave is non-zero exclusively at point A. In an analogue way, choosing p_- at K and p_+ at K$'$, one obtains an exclusive localization of Bloch weight at B. This results in an identical A/B localization pattern at K and K$'$.

In our model Hamiltonian, the valence bands of the trivial phase display a p_+ character at K and p_- at K$'$ (see Supplementary Fig. 2b). Hence, they exclusively contribute to the charge localization around point A. In the non-trivial phase, however, the valence bands do not share a common L_z eigenvalue at K and K$'$, leading to a charge delocalization across *both* points A and B. A similar argument has been developed by Koshino *et al.* for the charge localization in twisted bilayer graphene [40].

Wannier Model

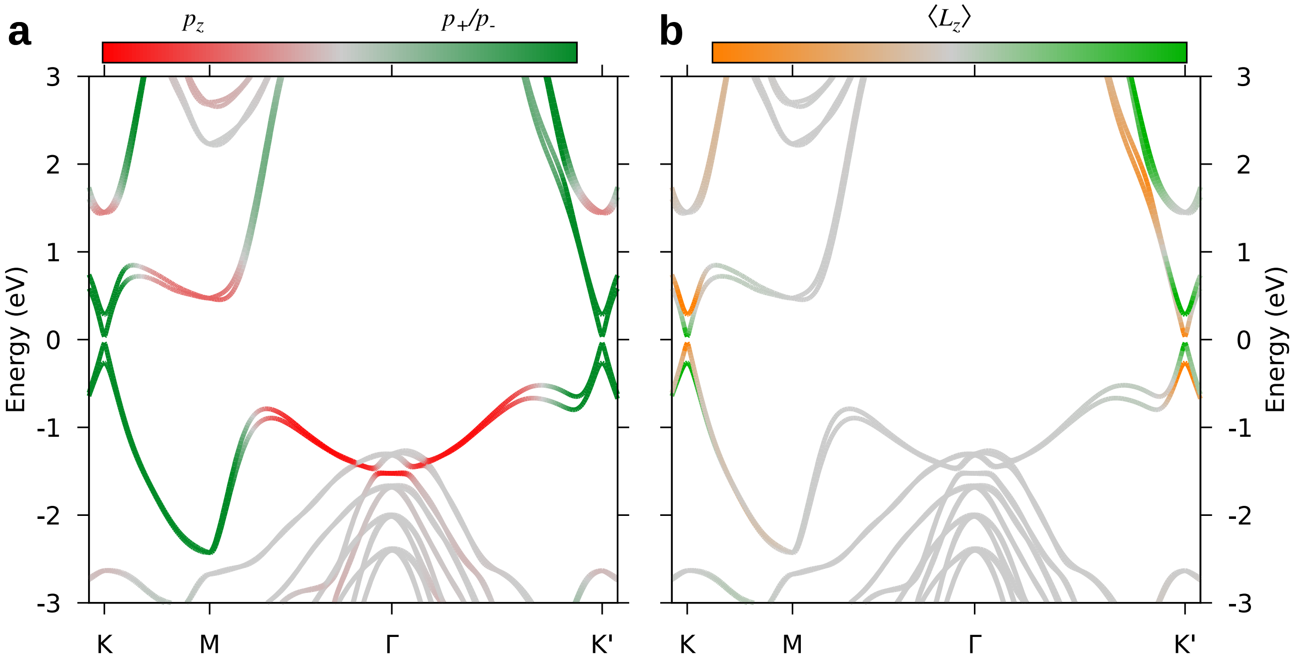

Supplementary Figure 3. **a**, Orbital character and **b** orbital angular momentum L_z from first principles. At K the valence (conduction) bands have opposite L_z-character which indicates the band inversion.

The minimal model presented in Sec. Supplementary Note 2 is constructed upon the key interactions of In on SiC. For investigating the In p subspace from first principles, we calculate the electronic ground state with VASP [41–44] by sampling the Brillouin zone on a $12 \times 12 \times 1$ regular mesh and by applying the HSE06 [45] exchange-correlation functional. Performing a projection of the Bloch wave function onto an In s, p and SiC sp_3 trial-basis with WANNIER90 [46] results in a good description of the indium low-energy bands without disentanglement and spread minimization. In the preserved In s, p and SiC sp_3 basis, we calculate the In p orbital character and the L_z-expectation value as presented in Supplementary Figure 3. The band structure is insulating with p_z and in-plane bands in valence around the Γ-point and the Brillouin zone margin, respectively. At the valley momenta the low energy bands have a strong in-plane character, with both positive and negative L_z character in conduction and valence indicating the non-trivial phase.

Calculation of the topological invariant

Here we calculate the $\mathbb{Z}_2$-invariant by following the approach proposed by Soluyanov and Vanderbilt [47]. Supplementary Fig. 4 shows the Wannier charge center (WCC) flow calculated with the Wannier Hamiltonian described in Sec. Supplementary Note 2. All non-flowing WCCs remain two-fold degenerate during the whole pumping cycle. Only the pair starting at $\overline{x} = 0.44$ splits-up, the largest gap intersects one time with an uneven number of WCCs indicating the non-trivial phase $\mathbb{Z}_2 = 1$. This jump occurs at $k = 0.33$, which comprises three degenerate WCC-pairs and the lower WCC branch of the moving WCC pair.

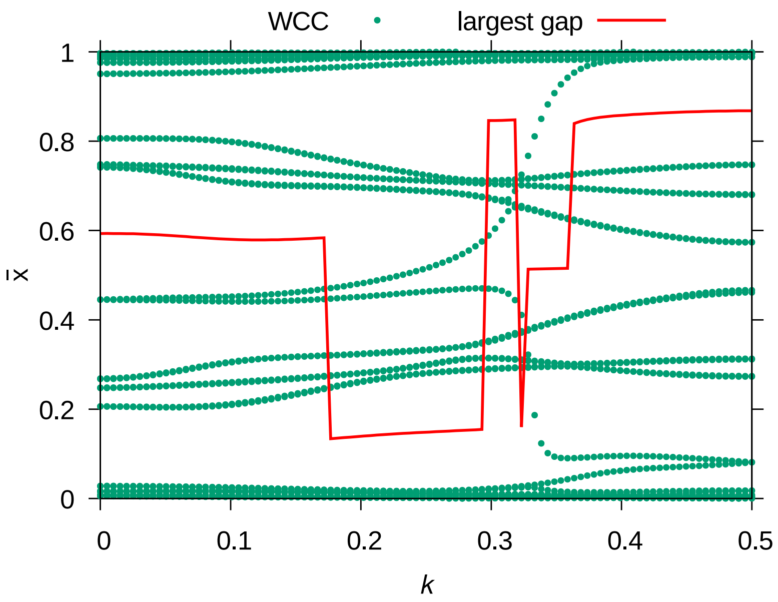

Supplementary Figure 4. Non-trivial WCC flow of indenene on SiC. The pumping momentum k and the WCCs $\overline{x}$ are given in reduced coordinates.

Supplementary Note 3. CHEMICAL AND STRUCTURAL CHARACTERIZATION

Chemical composition

The chemical composition of a representative indenene sample was investigated by X-ray photoelectron spectroscopy (XPS) in Supplementary Fig. 5. The spectrum shows the substrate and indium related core levels, but no sign of contaminants such as oxygen, indicating the high purity of the indenene layer.

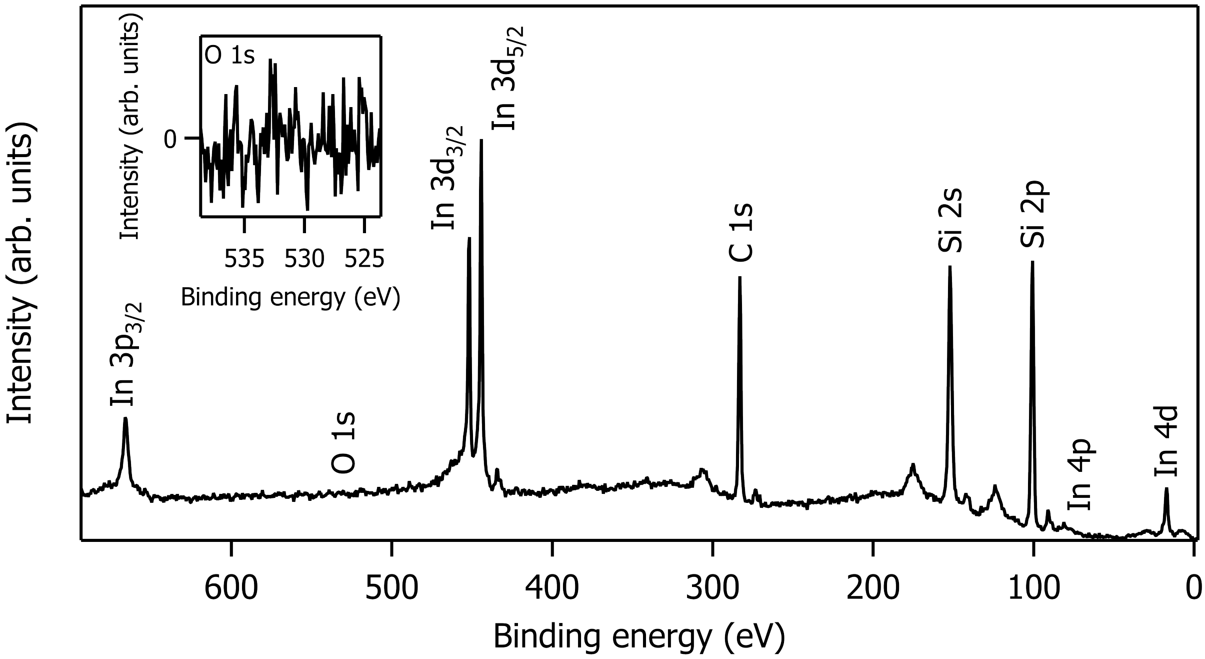

Supplementary Figure 5. XPS overview of a representative indenene sample, measured at room temperature with unmonochromatized Al-K_α radiation. The inset is a close-up of the oxygen 1s core level region, showing no trace of surface oxidation.

Crystalline surface lattice

As outlined in the main manuscript, indenene is grown by molecular beam epitaxy on a SiC(0001) substrate. The individual growth stages are monitored by low-energy electron diffraction (LEED) in Supplementary Fig. 6. Initially, we stabilize the hexagonal SiC(0001) (1×1) surface unit cell by hydrogen saturation of the SiC dangling bonds [48]. This yields a LEED pattern as depicted in Supplementary Fig. 6a. As the (1×1) LEED signature of the indenene film is identical to the signature of the unreconstructed hydrogen saturated substrate surface, they cannot be distinguished. We thus offer an excessive amount of indium and generate a high coverage phase of indium on SiC(0001). This intermediate film displays the distinct (7×7) surface reconstruction in Supplementary Fig. 6b that differs from bare SiC, and indicates the removal of hydrogen along with a successful growth of indium (further supported by scanning tunneling microscopy (STM) and XPS; not shown). Now, we remove the excessive indium by repeated annealing steps until the (1×1) LEED pattern in Supplementary Fig. 6c is recovered. XPS (discussed in more detail in Sec. Supplementary Note 3), angle-resolved photoelectron spectroscopy (ARPES) and scanning tunneling spectroscopy (STS) now reveal the distinct features of a monolayer of indium, i.e. indenene.

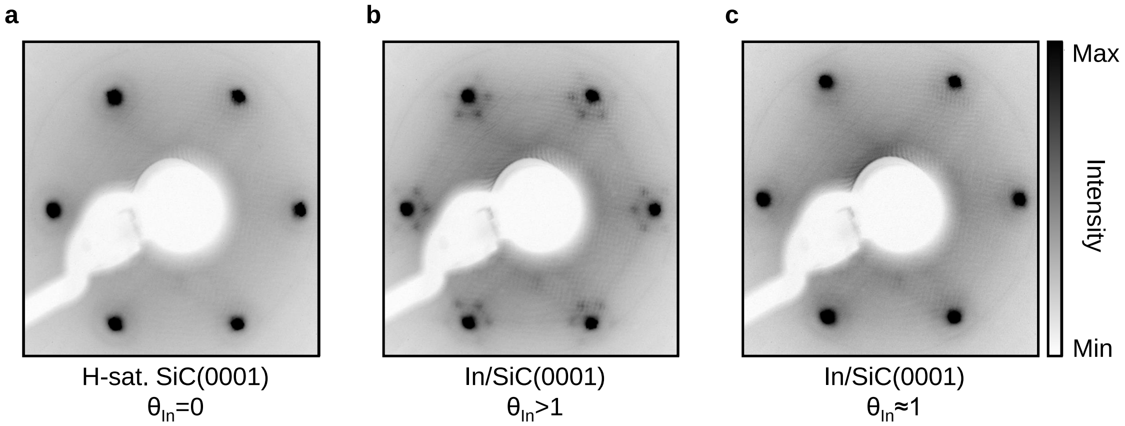

Supplementary Figure 6. Representative LEED pattern at 95 eV of **a**, the unreconstructed (1×1) surface of hydrogen etched SiC(0001); **b**, the (7×7) reconstruction of the high coverage phase of indium; and **c**, the (1×1) surface of the indium monolayer after annealing. Please note that no traces of the $(\sqrt{3} \times \sqrt{3})$R30° reconstructed SiC substrate are observed [3, 49, 50].

Supplementary Fig. 7a depicts a representative STM image recorded after the treatment described above. Single indenene domains cover terraces of the SiC(0001) substrate which are separated by half (0.5 nm) and full (1 nm) unit cell steps [51], see Supplementary Fig. 7b. Partially uncovered SiC appears as patches with less order in the indenene film or close to step edges. Close up views depicted in Supplementary Fig. 7c,d reveal the high quality of indenene on the 10 nm scale. Here, triangular defects, preliminarily attributed to subsurface dopant atoms [52], disturb the charge landscape as can be seen by the apparent height fluctuations in Supplementary Fig. 7c. Their influence on the local electronic structure is discussed more detailed in Sec. Supplementary Note 5.

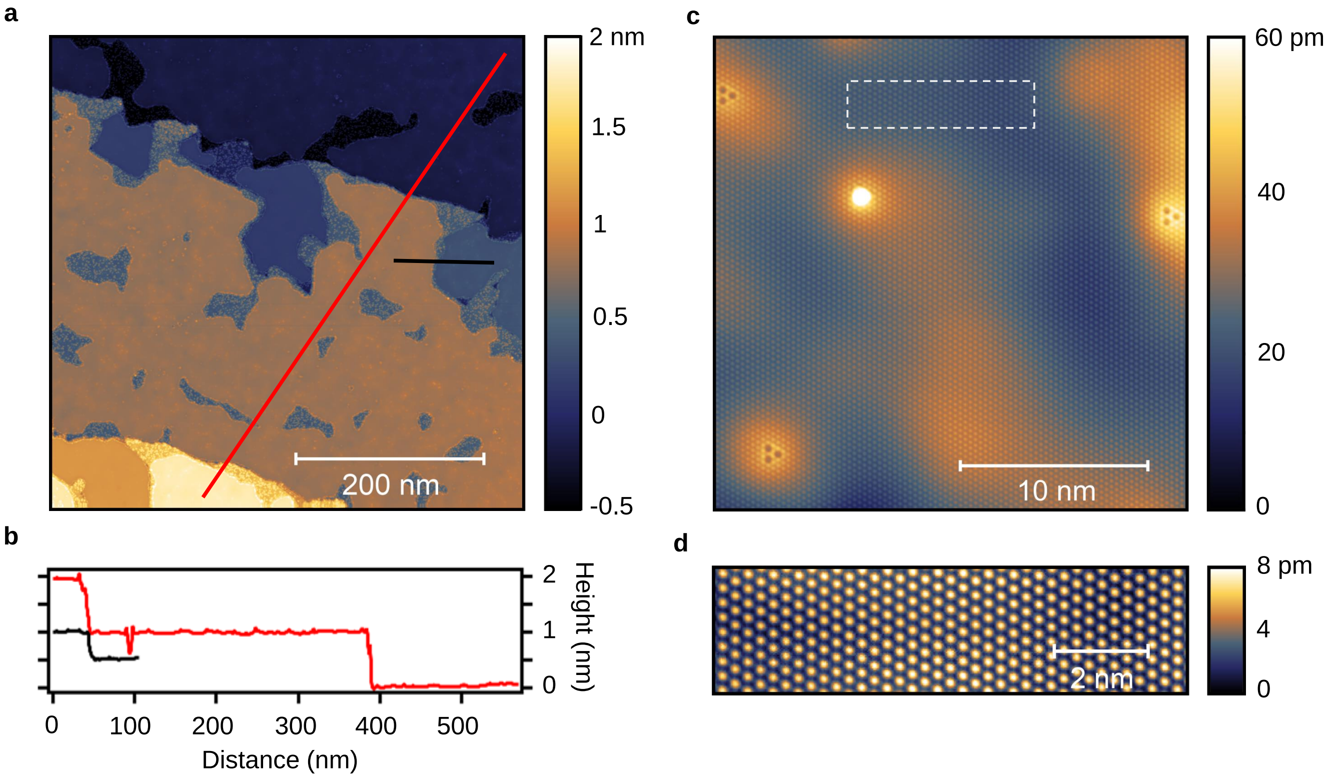

Supplementary Figure 7. Indenene topography at different length scales. **a** 500 nm × 500 nm STM scan acquired at a bias voltage of $V_{\text{set}} = 3\,\text{V}$ and tunneling current $I_{\text{set}} = 10\,\text{pA}$. **b** Line profiles extracted from **a** showing full (1 nm) steps (red) and a half (0.5 nm) step of the 4H-SiC(0001) unit cell. **c** 25 nm × 25 nm taken at $V_{\text{set}} = 500\,\text{mV}$ and $I_{\text{set}} = 150\,\text{pA}$. **d** 10 nm × 2.5 nm zoom in of the marked area in **c** exhibiting the triangular lattice of indenene.

Indium adsorption site

In order to confirm the T1 adsorption site of indenene on the SiC substrate, we study its topography by STM and support our observations by a detailed DFT relaxation study of indium centered at different adsorption sites.

At the experimental level, we investigate the relative lateral phase shift of the indium with respect to the silicon lattice along the $(11\bar{2}0)$ direction, crossing an edge separating indenene from the bare SiC substrate (c.f. Supplementary Fig. 8a and Fig. 3b of the main manuscript). On SiC, we achieve atomic resolution for a bias voltage of $\sim 1.65\,\mathrm{V}$ (see Supplementary Fig. 8a), while bias voltages inside the SiC band gap ($\sim 0.45\,\mathrm{V}$) are preferable for indenene (see Supplementary Fig. 8b). According to DFT, the tunneling current of the indenene film at 0.45 V is mostly produced by p_z orbitals oriented along the surface normal, allowing for a direct link between tunneling image and indium position. Earlier, DFT supported STM studies of the H-saturated SiC surface found an analogous relationship between the position of silicon atoms and their STM signature at 3.0 V bias voltage [48], a result that we find to be robust for the entire bias interval from 1.65 V to 3.0 V. Exploiting a defect feature that is common to both scans, we align the height profiles of the two differently biased topography scans in Supplementary Fig. 8c, and find a relative phase matching of the indium and the silicon lattice along $(11\bar{2}0)$. With T1 defined as the site above the silicon atom of the SiC(0001) surface, we conclude that indium adsorbs at the T1 site.

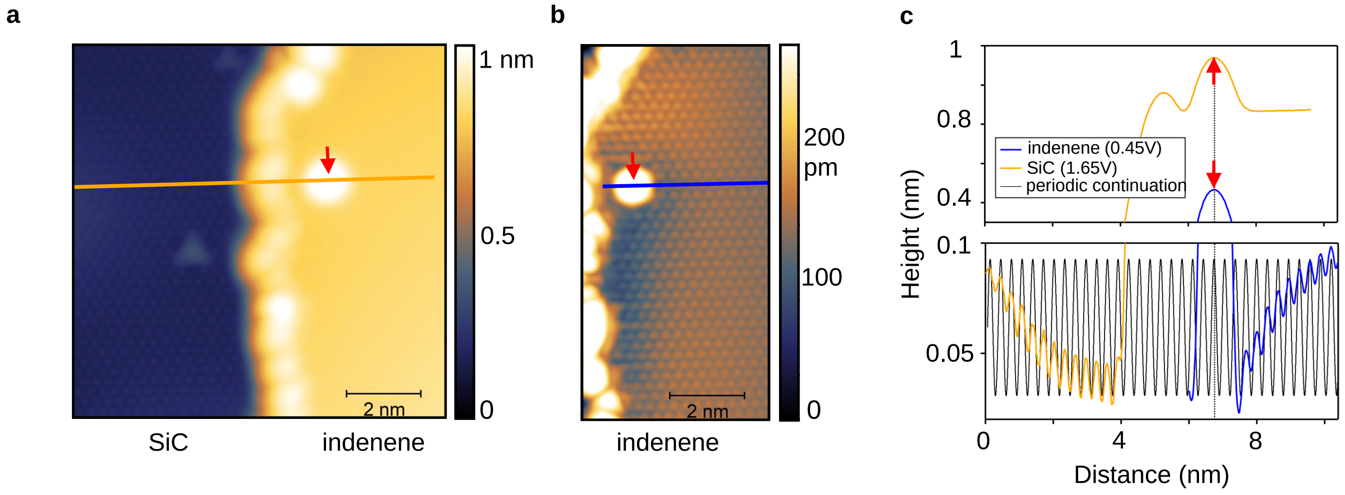

Supplementary Figure 8. **a**, STM topography scan across the SiC (left) to indenene (right) boundary, recorded with bias voltage $V_{\mathrm{set}} = 1.65\,\mathrm{V}$ and tunneling current $I_{\mathrm{set}} = 80\,\mathrm{pA}$. The defect feature is marked with a red arrow. **b**, Scan across the same indenene area as in **a**, this time measured with $V_{\mathrm{set}} = 0.45\,\mathrm{V}$ and $I_{\mathrm{set}} = 100\,\mathrm{pA}$ to obtain atomic resolution. **c**, Line profiles extracted from identical cuts along the orange line in **a** and the blue line in **b**. The lattice periodicity of SiC and indenene are in phase and indicate that both indium and silicon occupy the T1 site of SiC.

From STM scans across larger areas (c.f. Supplementary Fig. 7), and from a detailed LEED study in Ref. [53], we further identify the S2/S2* termination of the 4H-SiC(0001) substrate surface. On this basis, we now employ DFT in the generalized-gradient approximation as parametrized by Perdew-Burke-Ernzerhof [54] to relax indium on the 4H-SiC terminated substrate and to determine the indium adsorption site theoretically [41–44]. The total energy and the in-plane force acting on the indium atoms is calculated by sampling the indium in-plane coordinate on a 9×9 grid and relaxing the out-of plane coordinate. The total energy (calculated with respect to T1) and the normalized in-plane force exhibit a global minimum at the T1 position, confirming the experimental STM result. Additionally, we find a global energy maximum at the T4 site and a saddle point at H3, as shown in Supplementary Fig. 9 and summarized in Supplementary Table 2.

The T1 adsorption site of indium further becomes evident by analyzing the DFT band structures in Supplementary Fig. 10 and their comparison to our experimental ARPES result. First, the H3 and T4 positions would reduce the bonding distance between indenene and SiC as compared to T1. This would strengthen the impact of ISB and increase the ISB induced gap opening, thereby exceeding the gap induced by atomic SOC ($\approx 250\,\mathrm{meV}$), resulting in a topologically trivial insulator. This would contrast with the experimental charge localization pattern discussed in more detail in the main manuscript and Sec. Supplementary Note 5. Second, the H3 and T4 bonding scenario of indium on SiC would render a metallic system, in contrast to our ARPES observation of Dirac states located inside

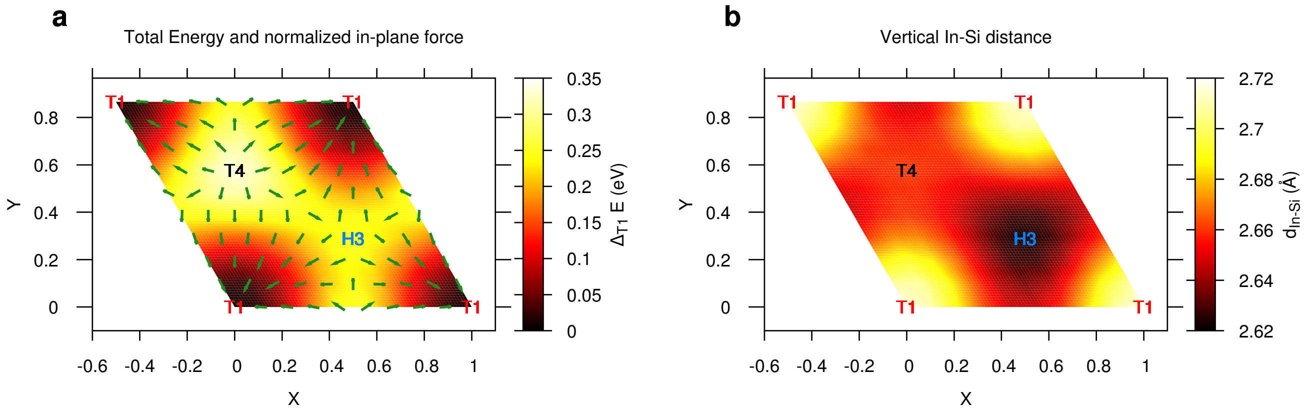

Supplementary Figure 9. DFT adsorption study of indium on the S2/S2* 4H-SiC terminated surface. **a** Total energy calculated with respect to its global minimum for indium adsorption at the T1 site. **b** Vertical distance between indium and surface silicon, giving a maximum distance for indium adsorption at T1. The in-plane x- and y-coordinates are given in units of the lattice constant.

a global band gap.

Site	$\Delta(E_0)$	$d_{\mathrm{In-SiC}}$	Δ_{global}	$\Delta_{\mathrm{K,ISB}}$
T1	-	2.72 Å	40 meV	224 meV
T4	340 meV	2.65 Å	-	1305 meV
H3	245 meV	2.62 Å	-	1595 meV

Supplementary Table 2. DFT (PBE) adsorption study of indium on Si terminated 4H-SiC (0001): Structural and electronic results are given for indium relaxed on the T1, T4 and H3 positions, respectively. The total energy $\Delta(E_0)$ is given with respect to the energy minium at the T1 site. $d_{\mathrm{In-SiC}}$ is the distance between the indium and SiC surface, Δ_{global} is the global band gap and $\Delta_{\mathrm{K,ISB}}$ is the ISB induced splitting at K/K′. As discussed in the text, the T4 and H3 adsorption sites relax into a metallic ground state.

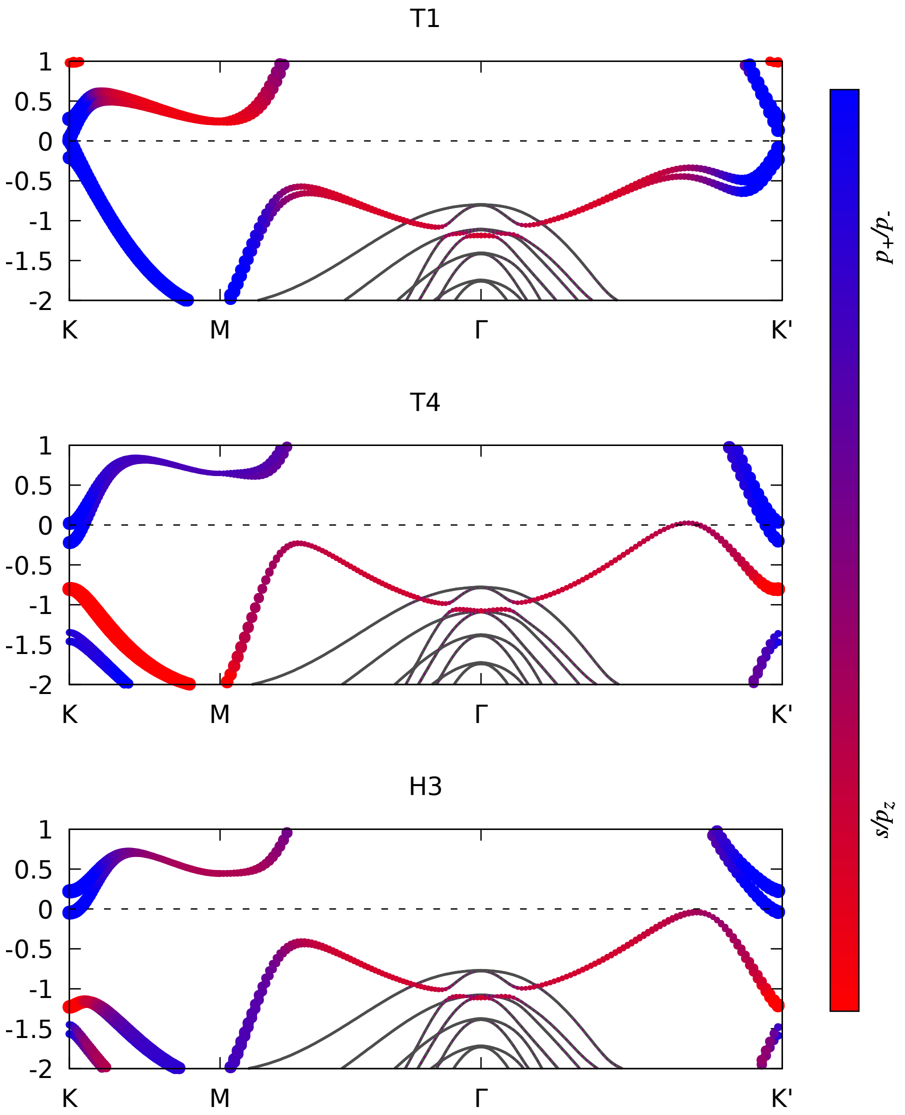

Supplementary Figure 10. Band structures of the relaxed DFT (PBE) unit cells with the indium atom on the T1, T4 and H3 site, respectively. The marker size denotes the indium character of the band, the marker color represents the orbital composition. The band structures for T4 and H3 are metallic, with trivial gap opening at K/K' due to strong ISB.

In-Si bond length

As argued in the manuscript and in Sec. Supplementary Note 3, the spacing between the indium layer and the SiC substrate is an important tuning parameter of indenene's topological character. We thus experimentally determine this distance by normal incidence X-ray standing wave (NIXSW) photo-emission experiments [55] at beamline I09 of the Diamond Light Source. The X-ray standing wave (XSW) is created by the interference of the incident X-ray beam with its Bragg reflection from the (0004) plane of SiC. For a given atomic species, the spatial intensity modulation of the standing wave field produces a photoelectron yield that depends on the position of the atomic emitters. Tuning the photon energy across the Bragg condition moves the standing wave throughout the top layers, producing a characteristic photon energy dependent line shape of photoemission intensity that depends on the alignment of the XSW and the atomic species under investigation.

Supplementary Fig. 11 summarizes the XSW results of indenene on SiC, showing the photoelectron yield measured for C 1s (blue), Si 2s (green) and In 4d (gray), as well as the corresponding X-ray reflectivity curve (red). By means of a standard fitting procedure of the XSW profiles [55], we extract the coherent position and the coherent fraction of the emitting atoms, i.e., their average vertical position and variation modulo the SiC (0004) lattice spacing. The resulting ambiguity in the positions is resolved by stipulating physically realistic bond lengths.

The average coherent positions and fractions obtained for indium, silicon and carbon are listed in Supplementary Table 3 for samples measured at room temperature (RT) and at low temperature (LT; $20-30\,\mathrm{K}$), respectively. The coherent positions estimated for silicon and carbon show that the bulk structure of SiC is retained close to the substrate surface. Electron-emission-angle-resolved XSW measurements further confirm the absence of surface relaxation effects.

From these results, we calculate the distance between the indium layer and the topmost silicon atoms as the difference in their coherent positions and find (2.669 ± 0.036) Å at RT and (2.671 ± 0.036) Å at LT, respectively, where the SiC(0001) bi-layer distance was taken from reference [51]. Both values are in good agreement with the relaxed bonding distance of 2.68 Å found by DFT (HSE06). The coherent fraction close to 1 determined for indium further reveals that the indenene film is highly ordered. Values for the coherent fraction above 1 are attributed to non-linear effects in the detector.

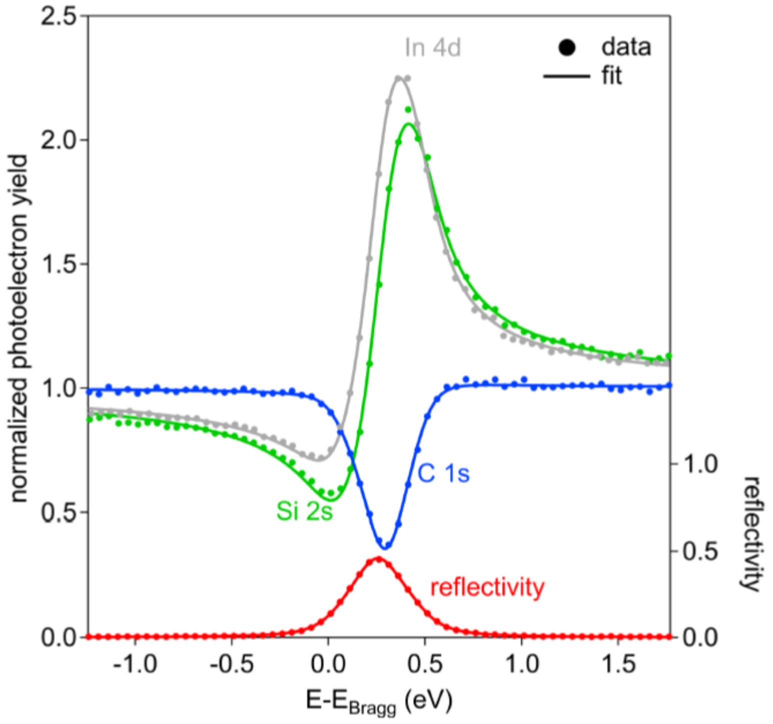

Supplementary Figure 11. X-ray reflectivity and NIXSW photoelectron yield profiles of indenene on SiC measured for photon energies tuned across the Bragg condition.

Element	RT (LT) Coherent position (Å)	RT (LT) Coherent fraction
Si	0.009 ± 0.025 (0.012 ± 0.025)	1.109 (1.085)
C	-0.623 ± 0.025 (-0.609 ± 0.025)	1.026 (1.006)
In	2.678 ± 0.025 (2.683 ± 0.025)	0.982 (1.016)

Supplementary Table 3. Average coherent positions and fractions of indium, silicon and carbon atoms. The absolute coordinates are calculated using the SiC(0001) bi-layer distance reported in reference [51].

Supplementary Note 4. BAND MAPPING

Substrate-induced carrier doping

The avoidance of charging effects in LT ARPES as well as STM/STS experiments on indenene requires the use of highly n-doped 4H-SiC(0001) substrates, which, however, increases the surface free charge carrier concentration and shifts the Fermi level into the conduction band. Estimating the charge carrier density n from the Fermi surface area of an assumed circular electron pocket centered at K, characterized by a Fermi wave vector of $k_F = (0.029 \pm 0.009)\,\text{Å}^{-1}$ (see Supplementary Fig. 12), we find $n \approx k_F^2/\pi = (2.7 \pm 1.7) \cdot 10^{12}\,\text{cm}^{-2}$ [56]. This value is of the same order of the room temperature sheet density of $n_{SiC} = (1.0 - 8.5) \cdot 10^{12}\,\text{cm}^{-2}$ extracted from the nominal specific resistance $\rho = (0.01 - 0.03)\,\Omega\text{cm}$ of the SiC substrate [57]. The metallicity of indenene can hence be attributed to a charge transfer from SiC. The error in k_F is calculated from the error of the circular fit as well as the instrumental resolution and contributions from local fluctuations of the chemical potential (see Sec. Supplementary Note 5).

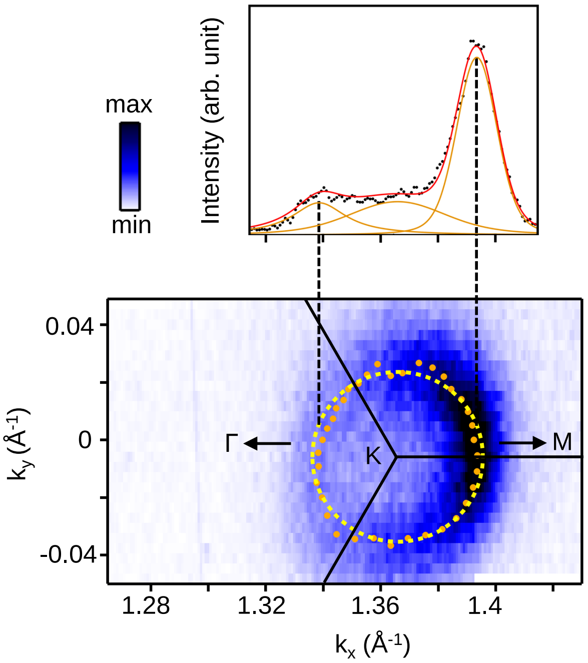

Supplementary Figure 12. Fermi surface electron pocket at the K point of indenene, measured with He-I radiation at 20 K and averaged over an energy window of 14.76 meV. MDCs cutting K at different angles were fitted with three Voigt peaks (orange) taking additional intensity from the second conduction band into account (see also Supplementary Fig. 13). Subsequently, a circle (yellow) was fitted to peaks corresponding to the first conduction band, from which we extract the size of the Fermi wave vector k_F.

Band gap determination at K/K′

To determine the size of the electronic band gap at K/K′, we present a series of ARPES cuts parallel to k_x, i.e., the ΓKM-direction in Supplementary Fig. 13a1-a5, sequentially spaced by $0.01\,\text{Å}^{-1}$ along the perpendicular k_y direction as marked by colored dashed lines in Supplementary Fig. 13d. The corresponding MDCs and energy distribution curves (EDCs) are depicted in Supplementary Fig. 13b1-b5 and Supplementary Fig. 13c1-c5, respectively. To extract the detailed band structure in proximity to K, the EDCs are fit by Voigt peaks accounting for the two sets of split valence and conduction bands, exemplified in Supplementary Fig. 13f. The fit results are summarized in Supplementary Fig. 13e, outlining the hyperbolic band dispersion of valence and conduction band, separated by a minimal gap of $\sim 125\,\text{meV}$ for a cut right through the K-point (red, Supplementary Fig. 13a3-c3).

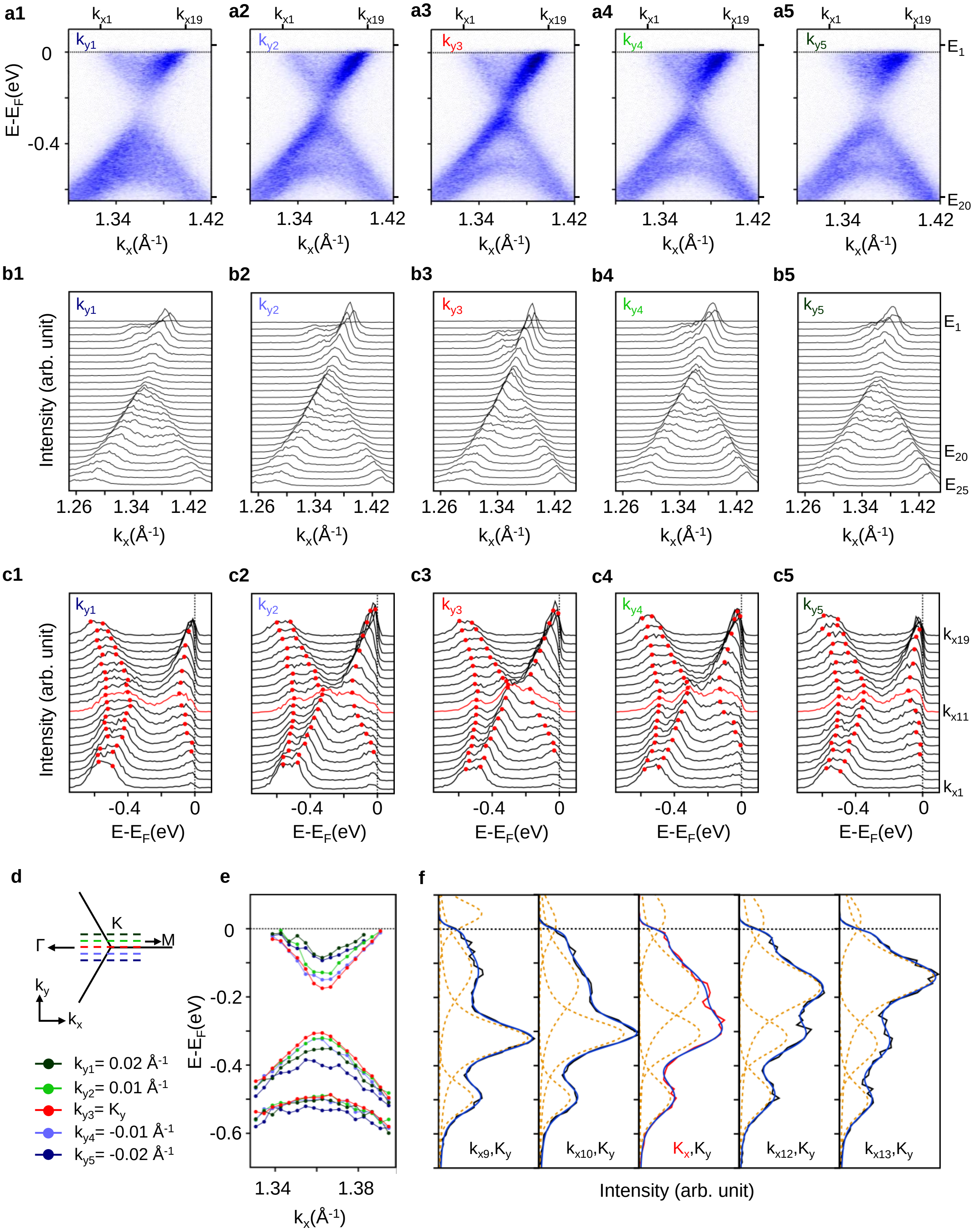

Supplementary Figure 13. **Band dispersion close to the K-point. a1-a5**, ARPES maps of indenene cutting the Dirac cone at (1) $k_y = -0.02$, (2) -0.01, (3) 0, (4) +0.01 and (5) +0.02 Å^{-1}. Corresponding MDCs and EDCs are shown in panels **b1-b5** and **c1-c5**, respectively. Red markers indicate the peak positions obtained by the Voigt fit. EDCs at $k_x = K_x$ are drawn in red. Colored lines in **d** mark the position of the cut with respect to the Brillouin zone. **e**, Fit result of the EDCs in **c**, showing the band dispersion close to the K-point. **f**, Voigt fit (dotted orange) and overall lineshape (blue) obtained for representative EDCs (black) in **c3**.

Supplementary Note 5. LOCAL CHARGE DISTRIBUTION AND SPECTROSCOPY

STS of degenerately doped semiconductors

Tunneling spectra taken on indenene show a finite differential conductance in the band gap, see, e.g., Fig. 3e of the main manuscript or Supplementary Fig. 14a (bottom). We attribute this offset to occupied conduction band (CB) states and their effect on tunneling spectroscopy. This experimental artifact is well documented in the literature and has been reported for other degenerately doped semiconductors like n- and p-type GaAs(110) [58], n-type InSb(110) [59], n-type ScN(001) [60], and n- and p-type H-terminated Si(100) [61, 62].

The underlying mechanism is described by a model for the tunneling current initially suggested by Feenstra *et al.* [58] which we adapt here to indenene by changing the gap value to 125 meV and assuming equal band masses for CB and valence band (VB). $I(V)$ and $\mathrm{d}I/\mathrm{d}V(V)$ curves are calculated for different shifts $\mathrm{E_S} = \mathrm{CBM} - \mathrm{E_F}$ of the semiconductor Fermi level $\mathrm{E_F}$ with respect to the conduction band minimum (CBM). The results are depicted in Supplementary Fig. 14b. Panel c and the blue curve represent the trivial situation with $\mathrm{E_F}$ located inside the band gap. No tunneling current flows at bias voltages V in the gap and thus $\mathrm{d}I/\mathrm{d}V$ is zero. However, if $\mathrm{E_F}$ is shifted into the CB, as illustrated in Supplementary Fig. 14d, a doping-induced current I_D tunnels into the tip, even though V is positioned inside the gap. Since the tunneling barrier depends on the applied bias voltage [58, 63], variations in V also change the barrier height "seen" by the occupied CB states that inevitably result in a finite differential conductance (gray curve). This offset increases for higher CB occupation as in the case of the red curve ($\mathrm{E_S} = 200\,\mathrm{meV}$), representing the Fermi level position observed in ARPES. The doping-induced contribution to $\mathrm{d}I/\mathrm{d}V$ is shown for lower bias voltages (dashed line). Clearly, the VB contribution exceeds the doping-induced fraction quite rapidly, allowing to neglect the former at lower bias voltages.

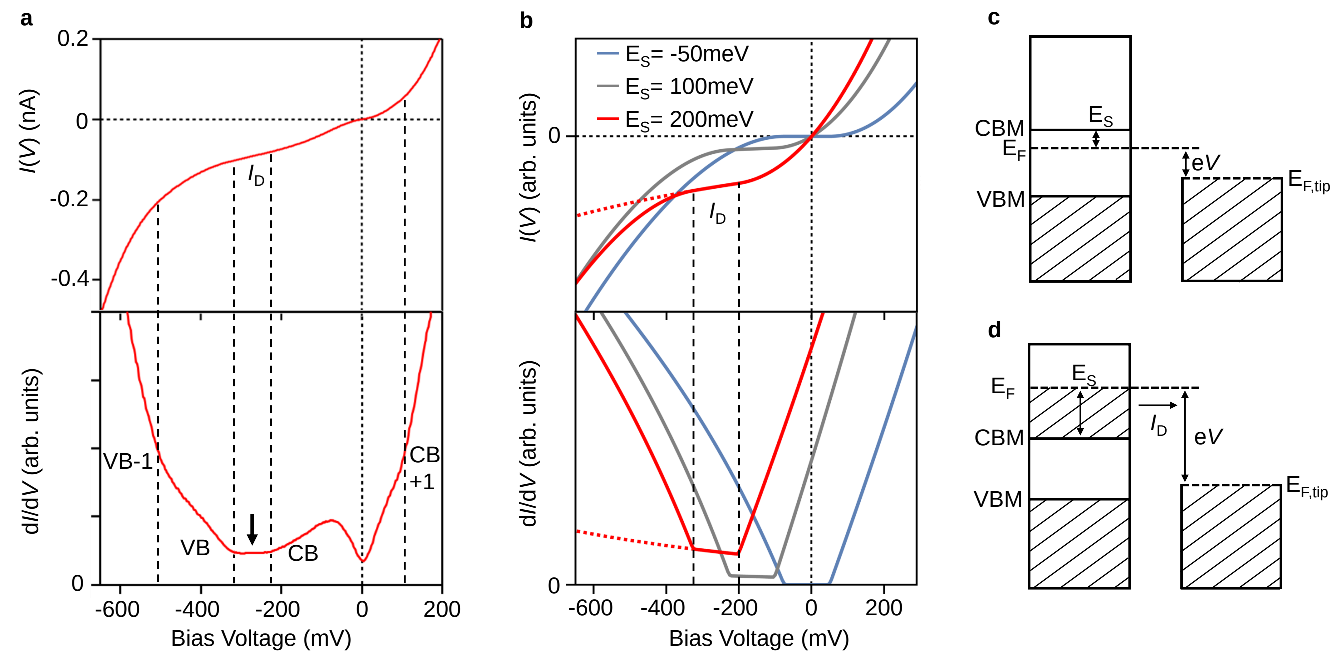

Supplementary Figure 14. **a**, Tunneling current $I(V)$ (top) and differential conductance $dI/dV(V)$ (bottom) taken on indenene. **b**, Model for the tunneling current adapted from Feenstra *et al.* [58] showing $I(V)$ (top) and $\mathrm{d}I/\mathrm{d}V(V)$ (bottom) spectra for different shifts $\mathrm{E_S} = \mathrm{CBM} - \mathrm{E_F}$. We change the gap value to 125 meV and assume equal masses for CB and VB. Band bending is neglected for simplicity. **c**, Schematic tunneling diagram of a semiconductor with the sample Fermi level $\mathrm{E_F}$ positioned in the gap. In the depicted situation the applied bias voltage $\mathrm{e}V = \mathrm{E_{F,tip}} - \mathrm{E_F}$ is located in the gap and thus no tunneling current is able to flow. $\mathrm{E_{F,tip}}$ is the Fermi level of the tip and VBM the valence band maximum. **d**, Tunneling diagram for a degenerately n-doped semiconductor with the Fermi level above the CBM. For bias voltages set in the gap the occupied CB states contribute the doping-induced tunneling current I_D.

Local fluctuations of the chemical potential

A STM topography scan across a large indenene monolayer and measured in constant current mode (CCM) is shown in Supplementary Fig. 15a. The scan unveils pronounced spatial variations that are neither related to sample height, nor to surface steps nor mechanical bending [64]. In fact, we attribute these variations to a spatial modulation in the surface potential, i.e., the Fermi level, produced by an inhomogeneous distribution of substrate doping on the nm scale. For indenene, this "apparent height" is particularly low close to film defects, marked by red arrows in Supplementary Fig. 15a.

As exemplified by representative tunneling spectra on the same surface in Supplementary Fig. 15b, we find the onset of the p_z-derived states (around ~ -800 mV and ~ 150 mV) to vary spatially with tip position, showing a correlation between spectra recorded at lower apparent height to also be shifted to higher binding energies.[1] A similar effect was found in Ref. 64 and attributed to tip-induced band bending, strongly influenced by the presence of subsurface dopants.

To provide an estimate of the nm-scaled Fermi level variation due to this inhomogeneous doping distribution, two representative spectra with extremal energy shifts are shown in Supplementary Fig. 15c. In analogy to a procedure applied to graphene [65], we now compare the energy position of a prominent minimum in the dI/dV curve (vertical lines), and find a shift of up to ± 20 mV across the entire scan area. This value is consistent with reported Fermi level fluctuations in bilayer graphene on SiC [52], and indicates a significant "smearing" of the Fermi level on the nm-scale.

Besides these local fluctuations, we further identify additional variations in the dI/dV-minimum when changing the tip position on a μm-scale, which are potentially related to changes in the dopant density between those regions. Considering both nm- and μm-scaled fluctuations, the measured spectra shift with respect to each other by ± 40 mV giving a measure of the overall Fermi level fluctuations that lower the energy resolution of macroscopic spectroscopy probes such as ARPES.

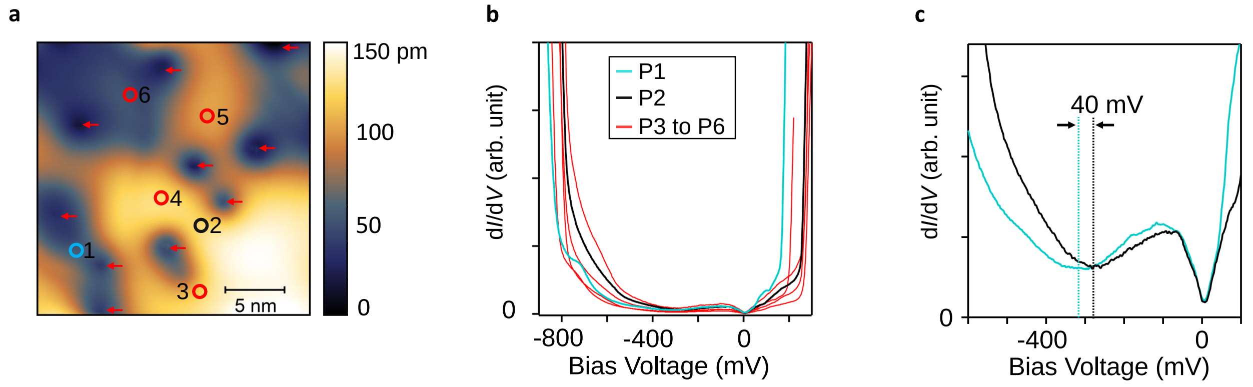

Supplementary Figure 15. **a**, Representative CCM scan of indenene measured with $V_{\mathrm{set}} = -850$ mV and $I_{\mathrm{set}} = 30$ pA, showing variations of the apparent height in the vicinity of defect structures marked with arrows. **b**, dI/dV spectra taken at different positions indicated by colored circles in **a**. Lower apparent heights correspond to regions where the spectrum is shifted non-rigidly towards higher binding energies. **c**, Close-up of the extremal spectra recorded at positions (1) and (2) in **a**. From the energy shift of a prominent minimum feature (vertical lines), we estimate a ± 20 mV Fermi level variation over nm-length scales. Depicted dI/dV-curves are the average of 100 single spectra taken at the respective positions indicated in **a**.

[1] Note that the set point height of all spectra has been explicitly readjusted by the height difference between the sites measured in Supplementary Fig. 15a to exclude z-dependent effects.

Charge localization as topological fingerprint in DFT

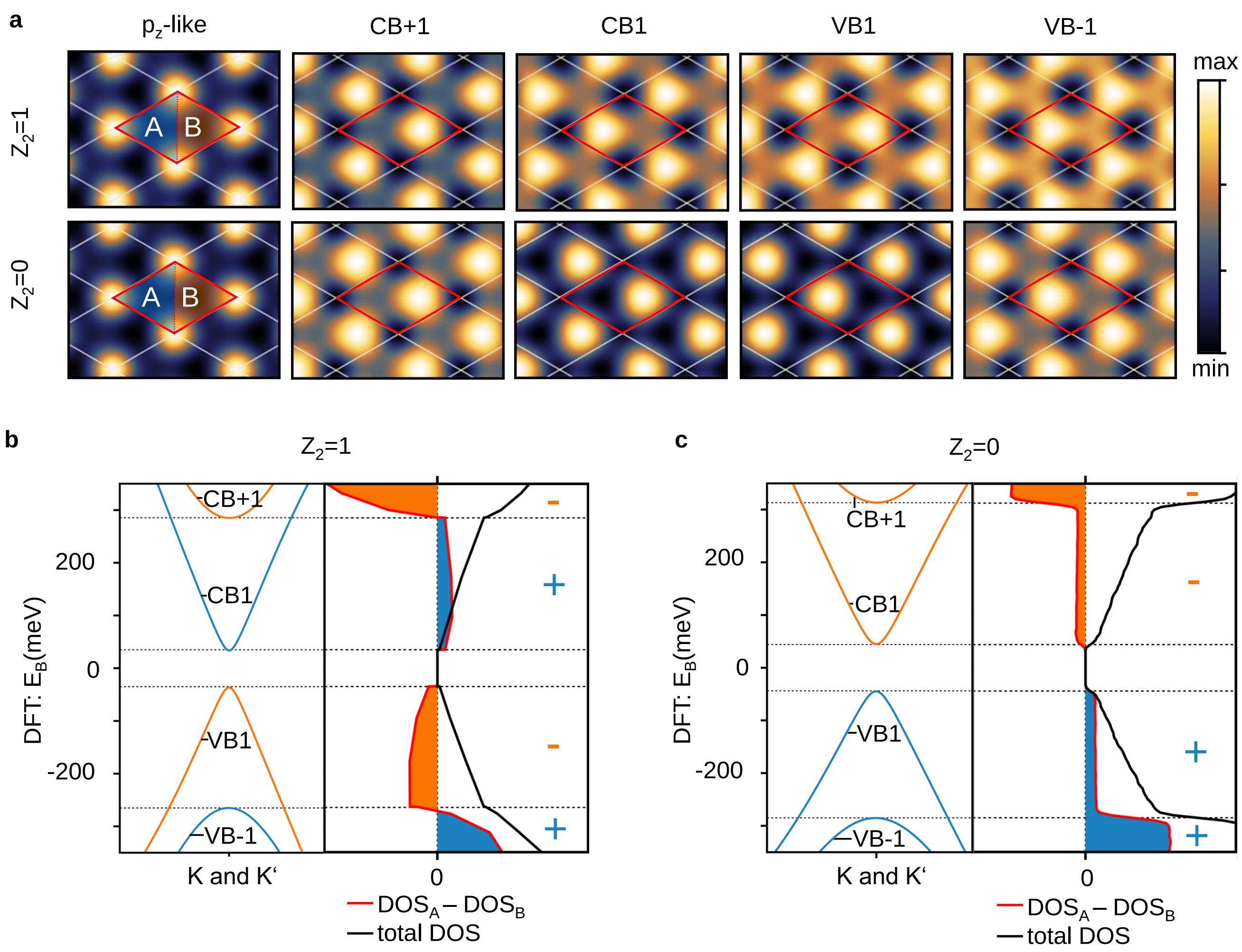

Supplementary Figure 16. **a**, Charge localization of the four different band contributions, calculated for two different silicon-indium bonding distances d: The top row shows the result of a fully relaxed surface with $d = 2.68$ Å, resulting in a topologically non-trivial state of indenene classified by a $Z_2 = 1$ invariant. The bottom row is calculated for a contracted value of $d = 2.5$ Å, increasing the impact of ISB and producing a topologically trivial phase: $Z_2 = 0$. Topological non-trivial and topologically trivial phases of indenene differentiate in their opposite charge localisation of the CB1 and VB1 within the unit cell. The leftmost simulation was calculated for energies dominated by p_z-derived states that mark the position of the indium atoms. Panels **b** and **c** show close-ups of the DFT band structure near the K-point, calculated for the topological non-trivial and trivial systems discussed in **a**. Additionally, we plot the total DOS as well the difference of DOS located at inequivalent positions A (blue) and B (orange) within the unit cell. While topological trivial indenene would show only a single sign change in the difference spectrum, topologically non-trivial indenene exhibits three sign changes.

Normalization of STS data

In this section, we discuss the normalization of the dI/dV difference spectra shown in the main manuscript and in Supplementary Fig. 18 which is used to minimize their dependence on the exponential part of the transmission function $T(r, z, E, eV)$ [63]. The energy E and position r dependent transmission function is part of the tunneling matrix element and mainly encodes the details of the particular tunneling junction, such as tip to sample distance z, the barrier height $\Phi(z)$ and the applied bias voltage V. As $T(r, z, E, eV)$ decays exponentially with z, a meaningful comparison of $\mathrm{d}I/\mathrm{d}V$ curves taken at varying z requires a careful rectification of this term.

Most commonly, $\mathrm{d}I/\mathrm{d}V$ is normalized by the voltage dependent conductance I/V, where the tunneling current $I(V)$ can be approximated as [63]

$$I(V) \propto e \int_0^{eV} \rho(r, E)T(r, z, E, eV)\mathrm{d}E \ . \tag{29}$$

Since $T(r, z, E, eV)$ is contained in both quantities, their ratio tends to cancel the exponential dependence. Thus, this procedure yields an almost z-independent term that is still proportional to the local density of states (LDOS) $\rho(r, E)$ which is of general interest for the interpretation of STS. In order to determine the dominant DOS contribution within the indenene unit cell at a given V, we perform $\mathrm{d}I/\mathrm{d}V$ measurements at constant height with atomic resolution. With z and the p-orbital contribution to $T(r, z, E, eV)$ remaining unchanged for two subsequent measurement positions $r = A$ and B within the unit cell, we can safely assume

$$T(A, z, E, eV) = T(B, z, E, eV) \equiv T(z, E, eV) \ , \tag{30}$$

from which we calculate the difference spectrum

$$(\mathrm{d}I/\mathrm{d}V)_A - (\mathrm{d}I/\mathrm{d}V)_B \propto e\ T(z, eV, eV)(\rho(A, eV) - \rho(B, eV)) \ , \tag{31}$$

shown in Supplementary Fig. 17a. To further reduce the dependence on $T(z, E, eV)$, we normalize expression 31 by the tunneling conductance I_{avg}/V averaged over both sites A and B and plot the respective result in Supplementary Fig. 17b.[2]

As outlined in Supplementary Fig. 17, both quantities can be interpreted as a qualitative measure of the energy dependent charge imbalance between site A and B. As predicted and discussed in more detail in Sec. Supplementary Note 5, we observe three distinct sign changes, marked by transitions from orange to blue regions, identifying the non-trivial topological character of indenene. The dotted lines hereby mark the energy positions where the difference spectra exceed the noise-level, and the sign change is statistically significant. This is especially relevant in the voltage-interval from $-180\,\mathrm{mV}$ to $-300\,\mathrm{mV}$ where the energy gap is expected. The dotted lines give a rough estimate of the gap size.

[2] Please note that an individual normalization of the $\mathrm{d}I/\mathrm{d}V$-spectra by site-specific I/V curves would lead to misleading site-dependent factors. They are due to the different LDOS contribution at A and B entering the energy integral of $I(V)$ in relation 29.

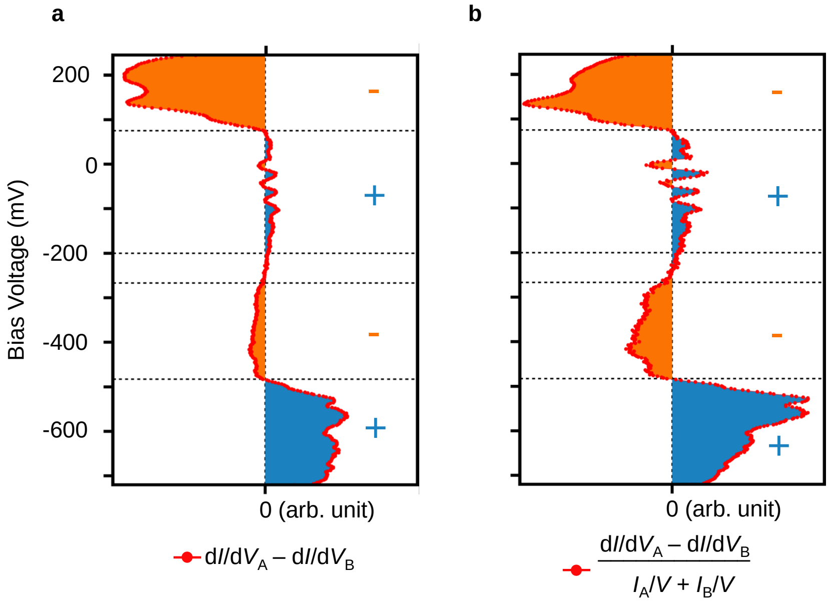

Supplementary Figure 17. **a**, Difference spectrum calculated according to expression 31. Blue (positive) indicates energy regions with the LDOS maximum located at position A; orange (negative sign) indicates energy regions where the LDOS maximum is located at position B. Dotted lines mark the relevant sign changes discussed in Fig. 5 of the main text. **b**, Difference spectrum from **a** normalized by the average tunneling conductance I_{avg}/V. The spectra contain at least 30 averaged $\mathrm{d}I/\mathrm{d}V$ curves per site A and B, respectively.

Influence of the tunneling resistance on the charge localization

A recent study has shown that a topological phase transition can in principle be triggered locally by the electric field between STM tip and sample [66]. To exclude this effect and to demonstrate the robust topological character of indenene, we recorded STS curves and $\mathrm{d}I/\mathrm{d}V$-maps at different distances z between tip and sample, thus varying the electric field across the tunnel junction. The results are summarized in Supplementary Fig. 18, showing differential conductance maps in panel a and difference spectra as described in Sec. Supplementary Note 5 in panel b. Technically, the exploitable z-range is restricted to approximately 1 Å, where upper and lower limit are defined by vanishing tunneling signal and the stability of the tunneling contact, respectively. For all z within this range, we find the charge localization behaviour expected for topologically non-trivial indenene, discussed in more detail in Sec. Supplementary Note 5. Also note that the sign change in the valence bands in Supplementary Fig. 18b is robust with z, indicating stable ISB band splitting at K/K′.

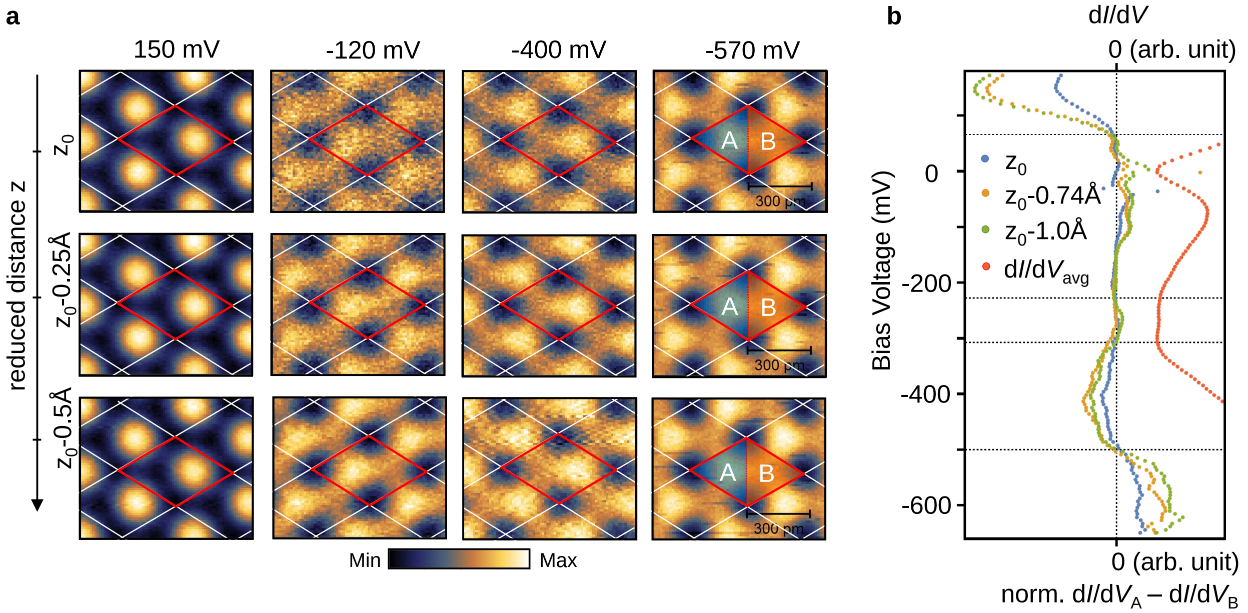

Supplementary Figure 18. **a**, Constant height mode differential conductance maps measured at different tip to sample distances z and for energies comparable to Fig. 5a. Except for the signal to noise ratio the DOS signature is robust in z. **b**, $\mathrm{d}I/\mathrm{d}V$ difference spectra between A and B site (see Sec. Supplementary Note 5). Significant sign changes are marked by dotted lines, and are robust with respect to z. Additionally, the spatially averaged $\mathrm{d}I/\mathrm{d}V$ signal (red) at $z_0 - 1.0$ Å is depicted. Difference spectra contain the average of 100 $\mathrm{d}I/\mathrm{d}V$ scans per site A and B, respectively.

[1] de Heer, W. A. *et al.* Large area and structured epitaxial graphene produced by confinement controlled sublimation of silicon carbide. *Proc. Natl Acad. Sci.* **108**, 16900–16905 (2011).

[2] Hamers, R. J. Effects of coverage on the geometry and electronic structure of Al overlayers on Si(111). *Phys. Rev. B* **40**, 1657–1671 (1989).

[3] Johansson, L., Owman, F. & Mårtensson, P. Surface state on the SiC(0001)-($\sqrt{3} \times \sqrt{3}$) surface. *Surf. Sci.* **360**, L478–L482 (1996).

[4] Vogt, P. *et al.* Silicene: Compelling experimental evidence for graphenelike two-dimensional silicon. *Phys. Rev. Lett.* **108**, 155501 (2012).

[5] Meng, L. *et al.* Buckled silicene formation on Ir(111). *Nano Lett.* **13**, 685–690 (2013).

[6] Fleurence, A. *et al.* Experimental evidence for epitaxial silicene on diboride thin films. *Phys. Rev. Lett.* **108**, 245501 (2012).

[7] Chiappe, D. *et al.* Two-dimensional Si nanosheets with local hexagonal structure on a MoS_2 surface. *Adv. Mater.* **26**, 2096–2101 (2014).

[8] Vitali, L., Ramsey, M. G. & Netzer, F. P. Trimer adatom structure of phosphorus on Ge(111). *Phys. Rev. B* **63**, 165320 (2001).

[9] Forti, S. *et al.* Mini-Dirac cones in the band structure of a copper intercalated epitaxial graphene superlattice. *2D Mater.* **3**, 035003 (2016).

[10] Kawazu, A. & Sakama, H. Geometric structure of the Si(111)$\sqrt{3} \times \sqrt{3}$-Ga surface. *Phys. Rev. B* **37**, 2704–2706 (1988).

[11] Dávila, M. E., Xian, L., Cahangirov, S., Rubio, A. & Lay, G. L. Germanene: a novel two-dimensional germanium allotrope akin to graphene and silicene. *New J.Phys.* **16**, 095002 (2014).

[12] Zhang, L. *et al.* Structural and electronic properties of germanene on MoS_2. *Phys. Rev. Lett.* **116**, 256804 (2016).

[13] Derivaz, M. *et al.* Continuous germanene layer on Al(111). *Nano Lett.* **15**, 2510–2516 (2015).

[14] Li, L. *et al.* Buckled germanene formation on Pt(111). *Adv. Mater.* **26**, 4820–4824.

[15] Olmstead, M. A., Bringans, R. D., Uhrberg, R. I. G. & Bachrach, R. Z. Arsenic overlayer on Si(111): Removal of surface reconstruction. *Phys. Rev. B* **34**, 6041–6044 (1986).

[16] Bringans, R. D., Uhrberg, R. I. G., Bachrach, R. Z. & Northrup, J. E. Arsenic-terminated Ge(111): An ideal 1×1 surface. *Phys. Rev. Lett.* **55**, 533–536 (1985).

[17] Rosenzweig, P. & Starke, U. Large-area synthesis of a semiconducting silver monolayer via intercalation of epitaxial graphene. *Phys. Rev. B* **101**, 201407 (2020).

[18] Lander, J. & Morrison, J. Surface reactions of silicon with aluminum and with indium. *Surf. Sci.* **2**, 553–565 (1964).

[19] Glass, S. *et al.* Triangular spin-orbit-coupled lattice with strong coulomb correlations: Sn atoms on a SiC(0001) substrate. *Phys. Rev. Lett.* **114**, 247602 (2015).

[20] Estrup, P. & Morrison, J. Studies of monolayers of lead and tin on Si(111) surfaces. *Surf. Sci.* **2**, 465–472 (1964).

[21] Ichikawa, T. & Ino, S. Structural study of Sn-induced superstructures on Ge(111) surfaces by RHEED. *Surf. Sci.* **105**, 395–428 (1981).

[22] Briggs, N. *et al.* Atomically thin half-van der Waals metals enabled by confinement heteroepitaxy. *Nature Materials* **19**, 637–643 (2020).

[23] Zhu, F.-f. *et al.* Epitaxial growth of two-dimensional stanene. *Nat. Mater.* **14**, 1020–1025 (2015).

[24] Deng, J. *et al.* Epitaxial growth of ultraflat stanene with topological band inversion. *Nat. Mater.* **17**, 1081–1086 (2018).

[25] Yuhara, J. *et al.* Large area planar stanene epitaxially grown on Ag(111). *2D Mater.* **5**, 025002 (2018).

[26] Park, C.-Y., Abukawa, T., Kinoshita, T., Enta, Y. & Kono, S. Low energy electron diffraction and X-ray photoelectron spectroscopy studies of the formation of submonolayer interfaces of Sb/Si(111). *Jpn. J. Appl. Phys.* **27**, 147–148 (1988).

[27] Okada, S., Kishikawa, Y., Oura, K. & Hanawa, T. LEED observation of the platinum induced superstructures on Si substrates. *Surf. Sci.* **100**, L457–L460 (1980).

[28] Okuda, T. *et al.* Surface core level shifts of the Au adsorbed Si(111) reconstructed surfaces. *J. Electron Spectrosc. Relat. Phenom.* **80**, 229–232 (1996).

[29] Howes, P. B., Norris, C., Finney, M. S., Vlieg, E. & van Silfhout, R. G. Structure of Ge(111)$\sqrt{3} \times \sqrt{3}$ R30°-Au determined by surface X-ray diffraction. *Phys. Rev. B* **48**, 1632–1642 (1993).

[30] Hilner, E. *et al.* Au wetting and nanoparticle stability on GaAs(111)B. *Appl. Phys. Lett.* **89**, 251912 (2006).

[31] Forti, S. *et al.* Semiconductor to metal transition in two-dimensional gold and its van der Waals heterostack with graphene. *Nat. Commun.* **11**, 2236 (2020).

[32] Lee, S. S. *et al.* Structural and electronic properties of thallium overlayers on the Si(111)-7×7 surface. *Phys. Rev. B* **66**, 233312 (2002).

[33] Hatta, S. *et al.* Atomic and electronic structure of Tl/Ge(111)-(1×1): LEED and ARPES measurements and first-principles calculations. *Phys. Rev. B* **76**, 075427 (2007).

[34] Seehofer, L., Falkenberg, G. & Johnson, R. STM study of the structure and phases of Pb on Ge(111). *Surf. Sci.* **290**, 15–25 (1993).

[35] Bihlmayer, G. *et al.* Plumbene on a magnetic substrate: A combined scanning tunneling microscopy and density functional theory study. *Phys. Rev. Lett.* **124**, 126401 (2020).

[36] Shioda, R., Kawazu, A., Baski, A. A., Quate, C. F. & Nogami, J. Bi on Si(111): Two phases of the $\sqrt{3} \times \sqrt{3}$ surface reconstruction. *Phys. Rev. B* **48**, 4895–4898 (1993).

[37] Ohtsubo, Y. *et al.* Structure determination of Bi/Ge(111)-($\sqrt{3} \times \sqrt{3}$)R30° by dynamical low-energy electron diffraction analysis and scanning tunneling microscopy. *J. Condens. Matter Phys.* **21**, 405001 (2009).

[38] Reis, F. *et al.* Bismuthene on a SiC substrate: A candidate for a high-temperature quantum spin hall material. *Science* **357**, 287–290 (2017).

[39] Slater, J. C. & Koster, G. F. Simplified LCAO method for the periodic potential problem. *Phys. Rev.* **94**, 1498–1524 (1954).

[40] Koshino, M. *et al.* Maximally localized Wannier orbitals and the extended Hubbard model for twisted bilayer graphene. *Phys. Rev. X* **8**, 031087 (2018).

[41] Kresse, G. & Furthmüller, J. Efficient iterative schemes for *ab initio* total-energy calculations using a plane-wave basis set. *Phys. Rev. B* **54**, 11169–11186 (1996).

[42] Kresse, G. & Joubert, D. From ultrasoft pseudopotentials to the projector augmented-wave method. *Phys. Rev. B* **59**, 1758–1775 (1999).

[43] Steiner, S., Khmelevskyi, S., Marsmann, M. & Kresse, G. Calculation of the magnetic anisotropy with projected-augmented-wave methodology and the case study of disordered $Fe_{1-x}Co_x$ alloys. *Phys. Rev. B* **93**, 224425 (2016).

[44] Blöchl, P. E. Projector augmented-wave method. *Phys. Rev. B* **50**, 17953–17979 (1994).

[45] Krukau, A. V., Vydrov, O. A., Izmaylov, A. F. & Scuseria, G. E. Influence of the exchange screening parameter on the performance of screened hybrid functionals. *J. Chem. Phys.* **125**, 224106 (2006).

[46] Mostofi, A. A. *et al.* Wannier90: A tool for obtaining maximally-localised Wannier functions. *Comput. Phys. Commun.* **178**, 685–699 (2008).

[47] Soluyanov, A. A. Computing topological invariants without inversion symmetry *Phys. Rev. B* **83**, 235401 (2011).

[48] Glass, S. *et al.* Atomic-scale mapping of layer-by-layer hydrogen etching and passivation of SiC(0001) substrates. *J. Phys. Chem. C* **120**, 10361–10367 (2016).

[49] Owman, F., Hallin, C., Mårtensson, P. & Janzén, E. Removal of polishing-induced damage from 6H-SiC(0001) substrates by hydrogen etching. *J. Cryst. Growth* **167**, 391 – 395 (1996).

[50] Mårtensson, P., Owman, F. & Johansson, L. I. Morphology, atomic and electronic structure of 6H-SiC(0001) surfaces. *Phys. Status Solidi B* **202**, 501–528 (1997).

[51] Stockmeier, M., Müller, R., Sakwe, S. A., Wellmann, P. J. & Magerl, A. On the lattice parameters of silicon carbide. *J. Appl. Phys.* **105**, 033511 (2009).

[52] Zhang, S., Huang, D., Gu, L., Wang, Y. & Wu, S. Substrate dopant induced electronic inhomogeneity in epitaxial bilayer graphene. *2D Mater.* **8**, 035001 (2021).

[53] Ando, H., Visikovskiy, A., Nakagawa, T., Mizuno, S. & Tanaka, S. Structure determination of hydrogen-terminated 4H-SiC(0001) by LEED. *Phys. Rev. B* **99**, 235434 (2019).

[54] Perdew, J. P., Burke, K. & Ernzerhof, M. Generalized gradient approximation made simple. *Phys. Rev. Lett.* **77**, 3865–3868 (1996).

[55] Woodruff, D. P., Cowie, B. C. C. & Ettema, A. R. H. F. Surface structure determination using X-ray standing waves: A simple view. *J. Phys. Condens. Matter* **6**, 10633–10645 (1994).

[56] Tadich, A. *et al.* Tuning the charge carriers in epitaxial graphene on SiC(0001) from electron to hole via molecular doping with $C_{60}F_{48}$. *Appl. Phys. Lett.* **102**, 241601 (2013).

[57] Baliga, B. J. *Wide Bandgap Semiconductor Power Devices Ch. 2* (Woodhead Publishing Ltd.-Elsevier, Sawston, 2019).

[58] Feenstra, R. M. & Stroscio, J. A. Tunneling spectroscopy of the GaAs(110) surface. *J. Vac. Sci. Technol. B* **5**, 923–929 (1987).

[59] Whitman, L. J., Stroscio, J. A., Dragoset, R. A. & Celotta, R. J. Scanning-tunneling-microscopy study of InSb(110). *Phys. Rev. B* **42**, 7288–7291 (1990).

[60] Al-Brithen, H. A., Smith, A. R. & Gall, D. Surface and bulk electronic structure of ScN(001) investigated by scanning tunneling microscopy/spectroscopy and optical absorption spectroscopy. *Phys. Rev. B* **70**, 045303 (2004).

[61] Pitters, J. L., Piva, P. G. & Wolkow, R. A. Dopant depletion in the near surface region of thermally prepared silicon (100) in UHV. *J. Vac. Sci. Technol. B* **30**, 021806 (2012).

[62] Fukutome, H. *et al.* Scanning tunneling microscopy study of the hydrogen-terminated n- and p-type Si(001) surfaces. *Appl. Surf. Sci.* **130**, 346–351 (1998).

[63] Feenstra, R., Stroscio, J. A. & Fein, A. Tunneling spectroscopy of the Si(111)2×1 surface. *Surf. Sci.* **181**, 295 – 306 (1987).

[64] Weidlich, P. H., Dunin-Borkowski, R. E. & Ebert, P. Quantitative determination of local potential values in inhomogeneously doped semiconductors by scanning tunneling microscopy. *Phys. Rev. B* **84**, 085210 (2011).

[65] Lauffer, P. *et al.* Atomic and electronic structure of few-layer graphene on SiC(0001) studied with scanning tunneling microscopy and spectroscopy. *Phys. Rev. B* **77**, 155426 (2008).

[66] Collins, J. L. *et al.* Electric-field-tuned topological phase transition in ultrathin Na_3Bi. *Nature* **564**, 390–394 (2018).
